# Surprise Parties

**Published:** 1866-05

**Authors:** 


### HALL'S JOURNAL OF HEALTH.

**Our Legitimate Scope is almost boundless: for whatever begets pleasurable and harmless feelings, promotes Health; and whatever induces disagreeable sensations, engenders Disease.**

WE AIM TO SHOW HOW DISEASE MAY BE AVOIDED, AND THAT IT IS BEST, WHEN SICKNESS COMES, TO TAKE NO MEDICINE WITHOUT CONSULTING A PHYSICIAN.

**Vol. XIII.] MAY, 1866. [No. 5.**

### SURPRISE PARTIES, are among the numerous underhand inventions of the "Adversary," as "Friends" term that wicked spirit, who, as a general rule, goeth about like a roaring lion seeking whom he may devour, but in this matter assumes the garb of philanthropy, as many of his followers in these latter days are prone to do. There is no objection to giving pleasant surprises to those whom we love, respect or admire, provided pernicious consequences do not result, legitimately and infallibly. The clergy in this country are the best men in it; they are the light of the world, the salt of the earth: for literary acquirements, for mental culture, for purity of morals and blameless lives, they have not their equals in any class of civilized society; and when such men devote their whole time to the preparation of books, essays, sermons and discourses for the instruction of the masses, encouraging them and persuading them to a life of purity, industry and thrift; warning them against whatever may deceive the head, corrupt the heart, debase the intellect, destroy the character and eventually ruin both body and soul; devoting themselves singly to these things, while others apply all their time and talents and energies towards making themselves, their children and their families, comfortable and happy, it is a very small matter that these last shall amply support the men, through whose influence, examples and teachings, their possessions are secured to them, and their rights, liberties and lives are preserved intact, day and night for years together, from the depredations of thieves and burglars and lawless, murderous men; for no man of thought can be so blind, as not to see, that if Bible teachings were to cease, and the Sabbath abolished, the whole foundations of society would be upturned; anarchy would ensue, and our streets run with human gore; revolutionary France proved all this; and who does not know, that where there is no preaching, and no Sunday, there springs up drunkenness and profanity, prostitution, social disorganization and every other evil work? The merchant pays his private watchman for guarding his property every night; the whole of the minister's time is expended in enforcing those precepts which, and which only, can make, not only property, but even life itself, secure in any community. The Broadway merchant or the Wall street broker or the South street shipper, would crimson with shame to have it known that his faithful night-watch had starved to death, on the pitiful salary which he had accorded him; and yet there are rich men and women, who give so little towards the support of the clergyman of the neighborhood, that he would actually starve if others did no bettter by him. The minister of any community has a right to demand an ample support, a salary large enough, regular enough, prompt enough, and sure enough, to enable him to have a mind at perfect ease in a pecuniary point of view; so that his undivided energies may be given to his proper work; that much he ought to have as a salary and no more; if that much is regularly and promptly paid, a surprise party is not needed; if that much is not accorded of right, then a surprise party, a donation party, and all similar inventions of that long-headed evil one, are underhand efforts to cripple the ministers in the long run; and like all underhand things, are mean in their very nature. In fact, these devices of the enemy are acknowledgments that the minister is not well enough paid, and that his people know it; and by these parties they seek to accord him as a favor, what belongs to him as a right; is there not here a palpable want of magnanimity? Do you wish your minister to have a feeling that he is under obligations to you for your contributions to these parties? If he knows where that handsome present comes from, is it in human nature for him to be as faithful to you in his reproof, as he ought to be? to feel as independent of your good will as he ought to do? Be assured, it is impossible. These parties are tacit bribes; they cannot but have to a greater or less extent, the effects of a bribe; but a minister's palm should be as clear of a bribe as that of a judge. Who would dare to bribe his judge? none but the meanest of his kind! These parties are fitful and uncertain; their tendency always is to make the people feel that their pastor's income is larger than it really is, because the results of such operations are always exaggerated. Of all things, uncertainty in the amount of salary is the most harrassing to a cultivated mind, it makes an immense difference in a family's happiness. It may be ventured as a truth, that a certain salary of a thousand dollars a year, punctually and cheerfully paid, gives more happiness to any family, than double the amount promised and merely possible, and at best, most uncertain. A paragraph is going the rounds, most applaudingly, that a clergyman had his rent increased one-half, and that as soon as his people heard of it, they promptly made him a present of that increase. A present! a beautiful thought; splendid idea; why not make it a generous deed, by adding that much to his salary! and then he would have no misgiving as the year closes, about its being made up to him again; would he not be more able to lay down the law and the testimony without fear, favor or affection; less likely to preach peace, when there was no peace, if he stood upon the higher ground of receiving a sufficient salary as a matter of right, not favor? There is another radical objection to these chance additions to the minister's salary. All persons who rely upon what is called chance, are demoralized, as beggars, gamblers, hunters, wreckers and raiders. Men who get a living by uncertain fees, such as lawyers, physicians and the like, are not reliable providers for their families, as a general rule; they are liberal only by fits and starts.

That people will be best fed from Sabbath to Sabbath whose godly minister is kept easy in his pecuniary matters, who has an income sufficient, if well managed, to meet his moderate wants; and it will continue as long as human nature remains as it is. "The laborer is worthy of his hire," said the master; nor should the sun go down on his wages; those wages should be equal to his comfortable support and should be paid to him without peradventure, always and in full, as his bounden right and just due; thus being generously supported by a loving people, he will be saved those health destroying anxieties which have many a time eaten out the lives of some of the best men ever known and laid them in a premature grave, to the great loss of the church, the community, and the world at large.

